# Comparison of optical coherence tomography-guided and intravascular ultrasound-guided rotational atherectomy for calcified coronary lesions

**DOI:** 10.1186/s12872-021-02103-5

**Published:** 2021-06-11

**Authors:** Weili Teng, Qi Li, Yuliang Ma, Chengfu Cao, Jian Liu, Hong Zhao, Mingyu Lu, Chang Hou, Weimin Wang

**Affiliations:** Department of Cardiology, Peking University People’s Hospital, NO.11 Xizhimen South Street, Xicheng District, Beijing, 100044 China

**Keywords:** Optical coherence tomography, Intravascular ultrasound, Calcified lesions, Rotational atherectomy

## Abstract

**Background:**

To compare the effect and outcomes of optical coherence tomography (OCT)-guided rotational atherectomy (RA) with intravascular ultrasound (IVUS)-guided RA in the treatment of calcified coronary lesions.

**Methods:**

Data of calcified coronary lesions treated with RA that underwent OCT-guided or IVUS-guided from January 2016 to December 2019 at a single-center registry were retrospectively analyzed. The effect and outcomes between underwent OCT-guided RA and IVUS-guided RA were compared.

**Results:**

A total of 33 lesions in 32 patients received OCT-guided RA and 51 lesions in 47 patients received IVUS-guided RA. There was no significant difference between OCT-guided RA group and IVUS-guided RA group in clinical baselines characteristics. Comparing the procedural and lesions characteristics of the two groups, the contrast volume was larger [(348.8 ± 110.6) ml vs. (275.2 ± 76.8) ml, *P* = 0.002] and the scoring balloon was more frequently performed (33.3% vs. 3.9%, *P* = 0.001) after RA and before stenting in the OCT-guided RA group. Comparing the intravascular imaging findings of the two groups, stent expansion was significantly larger in the OCT-guided RA group ([82 ± 8]% vs. [75 ± 9]%, *P* = 0.001). Both groups achieved procedural success immediately. There were no significantly differences in the incidence of complications. Although there was no statistical difference in the occurrence of MACE at 1 year between OCT-guided RA group and IVUS-guided RA group (3.1% vs. 6.4%, *P* = 0.517), no cardiovascular death, TVR and stent thrombosis occurred in OCT-guided RA group.

**Conclusions:**

OCT-guided RA compared to IVUS-guided RA for treating calcified coronary lesions resulted in better stent expansion and may have improved prognosis.

## Background

The treatment of coronary artery calcified lesions remains a serious challenge for percutaneous coronary intervention (PCI). Severely calcified lesions can lead to a higher prevalence of equipment delivery failure, stent under-expansion, and subsequent thrombosis and restenosis [1–3]. Rotational atherectomy (RA) is effective to modify calcified plaque to facilitate crossing calcified coronary lesions with any device and improve the success rate of procedure [4]. However, it is difficult to accurately assess the severity of calcified lesions, the efficacy of calcium modification and stent expansion based on coronary angiography. Intravascular ultrasound (IVUS) imaging is often used to assess coronary stent implantation and guide percutaneous coronary intervention, and IVUS-guided PCI can result in better clinical outcomes compared to angiography-guided PCI [5, 6]. The extent of calcification can be graded in IVUS images. However, ultrasound itself cannot penetrate calcium, due to acoustic shadowing, which limits the value of IVUS in the assessment of heavily calcified lesions. Optical coherence tomography (OCT) provides greater resolution imaging than IVUS and can in many cases assess calcium thickness and volume [7, 8]. The comparative of OCT-guided RA and IVUS-guided RA for calcified coronary lesions remains poorly studied. Thus, the aim of this study was to compare the effect and outcomes of OCT-guided RA with IVUS-guided RA in patients with coronary calcified lesions undergoing PCI.

## Methods

### Study design and subjects

This was a retrospective, single-center, observational study of patients with moderate or severe calcified lesions to compare the effect and outcomes of OCT-guided RA versus IVUS-guided RA. Between January 2016 to December 2019, patients with moderate or severe calcified lesions treated with RA under OCT guidance or IVUS guidance were enrolled. Patients were excluded from the study for the following: previous PCI and presence of in-stent restenosis; lesions with chronic total occlusions or bypass graft failure; insufficient image quality or intravascular image deficiency. Although the intravascular imaging devices used were at operator’s discretion, OCT was less likely to be used in patients with bifurcation lesions, ostial lesions, and renal insufficiency. All lesions were divided into OCT-guided RA group and IVUS-guided RA group based on the performance of OCT or IVUS image. The indication for RA was lesions with > 180° arc of calcium assessed by intracoronary imaging or lesions with angiographic moderate or severe calcification where an imaging catheter could not pass through. The study was conducted in accordance with the principles of the Declaration of Helsinki, and the study protocol was approved by the ethics review board of Peking University People’s Hospital. Informed consent was waived due to the retrospective nature of the study.

### Procedural details

All patients received an oral loading dose of 300 mg aspirin and 300 mg clopidogrel 12 h prior to the intervention procedure followed by maintaining dose of 100 mg aspirin and 75 mg clopidogrel once daily at least 1 year. Immediately before intervention, all patients received a bolus injection of heparin at a dose of 70–100 U/kg to maintain the activated coagulation time (ACT) > 300 s. Coronary angiography (CAG) was performed according to conventional standards. RA was performed by using the Rotablator™ RA System (Boston Scientific, USA) for lesion atherectomy in all cases. All the burr size included 1.25 mm, 1.5 mm, 1.75 mm, and 2.0 mm. The conventional 0.014-in. guidewire was replaced with the 0.009-in. ROTAWire™ Floppy guidewire (Boston Scientific, USA). The initial burr size was chosen based on the preprocedural intravascular imaging findings. When intravascular imaging catheter could not pass the lesions, the burr size was selected to reach a burr/vessel ratio of 0.4–0.6.

**Fig. 1:**
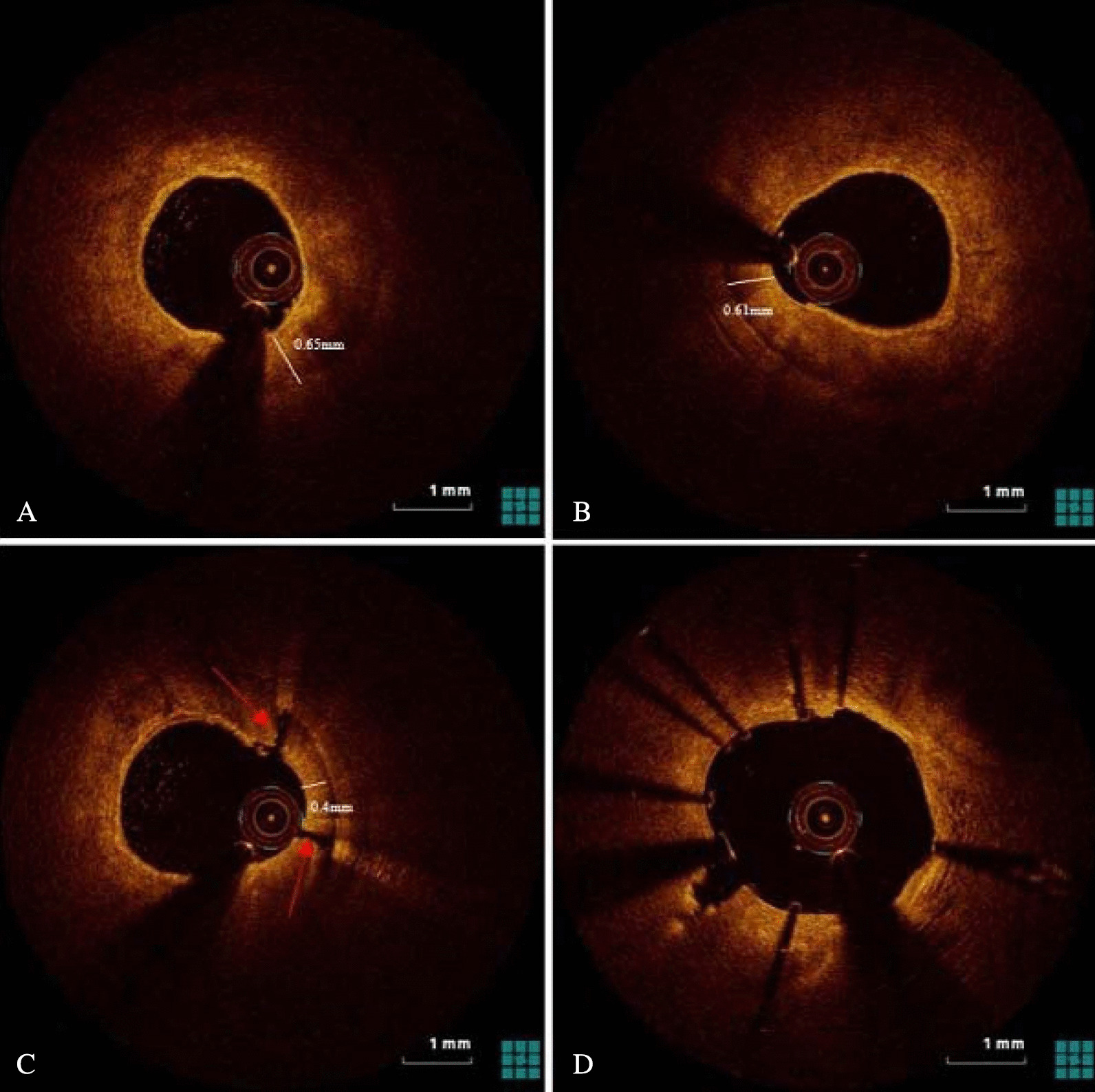
Representative one case of optical coherence tomography (OCT) guided rotational atherectomy (RA) images. **A** Pre-PCI OCT images showed heavily calcified plaque with a 360° calcium arc and a minimal calcium thickness of 0.65mm. **B** After RA with a 1.5-mm burr, the calcium thickness measured 0.61mm without crack formation. The operator decided to upsize the burr. **C** After additional atherectomy with a 1.75-mm burr, the calcium thickness became 0.40mm with cracks formation. **D** After stent implantation with post-dilatation OCT images showed adequate stent expansion (86%)

**Fig. 2:**
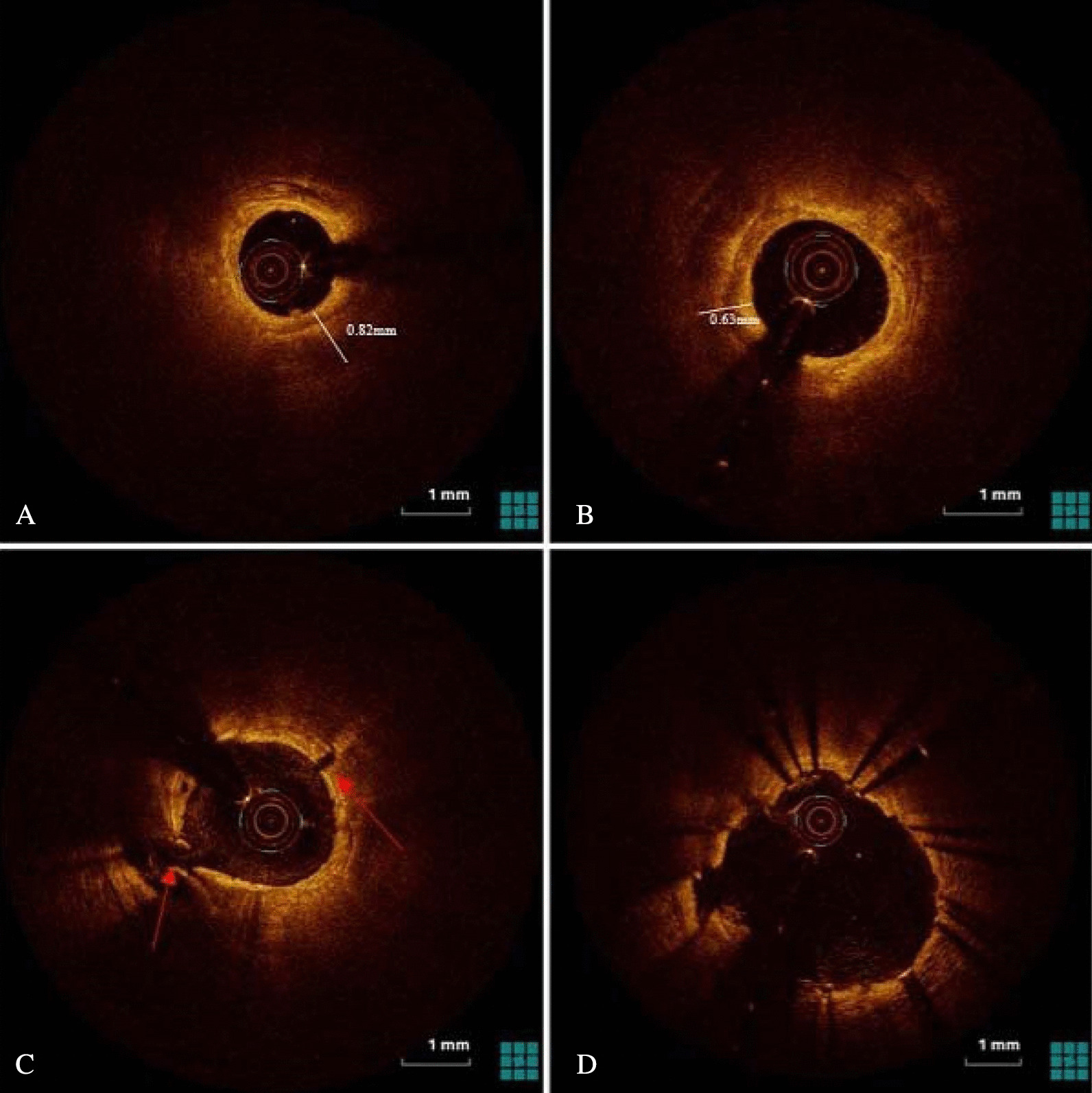
Representative another case of optical coherence tomography (OCT) guided rotational atherectomy (RA) images. **A** Pre-PCI OCT images showed heavily calcified plaque with a 360° calcium arc and a minimal calcium thickness of 0.82 mm. **B** After RA with a 1.75-mm burr, the minimal calcium thickness measured 0.63mm without crack formation. Subsequently, the operator performed a scoring balloon for dilation rather than upsize the burr. **C** After dilation with a 3.0 mm × 13 mm scoring balloon, OCT images showed disruption and cracks formation. **D** After stent implantation with post-dilatation OCT images showed adequate stent expansion (91%)

**Fig. 3:**
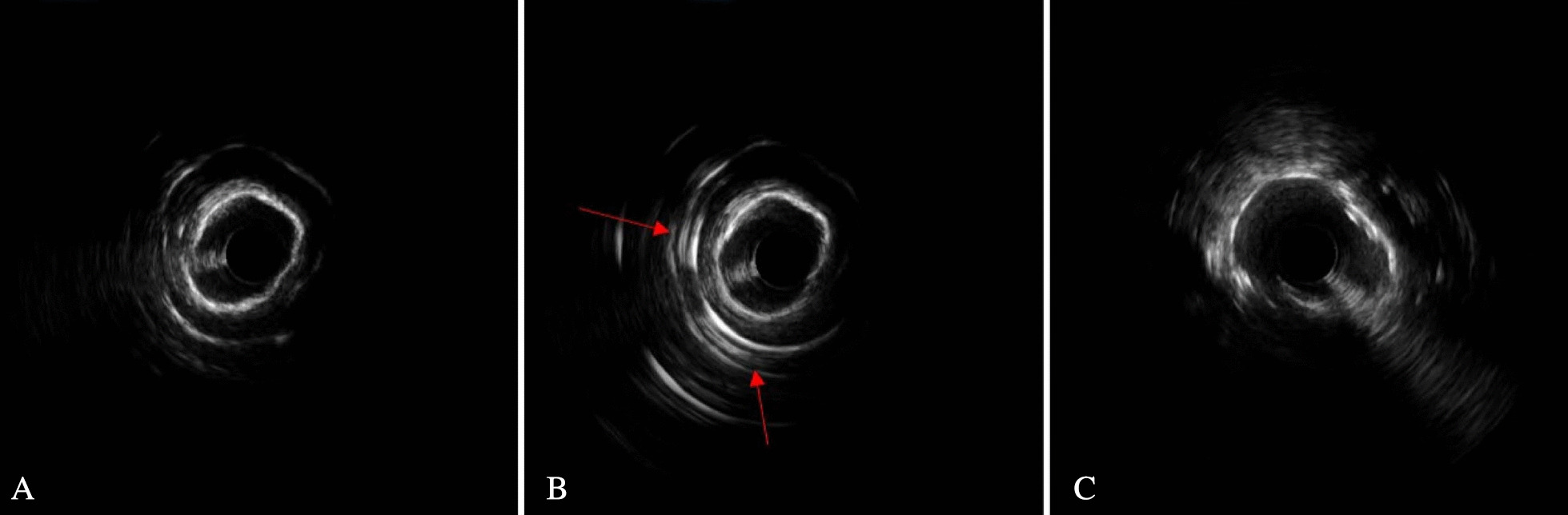
Representative one case of intravascular ultrasound (IVUS) guided rotational atherectomy (RA) images. **A** Pre-PCI IVUS images showed heavily calcified plaque with a 360° calcium arc. **B** After RA with a 1.5-mm burr, IVUS image showed reflection behind the calcification. The phenomenon usually was considered that the calcium was not thick. **C** After stent implantation with high pressure post-dilatation, IVUS images still showed stent under-expansion (73%)

Burr speed ranged from 140,000 to 180,000 rotation per minute. Each RA time was 10–15 s. During RA, the fluid containing unfractionated heparin and nitroglycerin was continuously injected intracoronary. After atherectomy, we performed intravascular imaging again to evaluate the efficacy of calcium modification and decided whether burr upsizing or balloon pre-dilatation was performed. The Lacrosse NSE ALPHATM (Goodman, Japan) is used scoring balloon. The type of the balloon was selected at operator’s discretion based on angiography and intravascular imaging. We performed a final intracoronary imaging after stent implantation with subsequent post-dilatation to evaluate the immediate effect of stent implantation. Figures 1, 2 and 3 showed the representative cases of OCT and IVUS guided RA images.

### Intracoronary image acquisition and analysis

Both OCT and IVUS of each imaging pullback were performed after 200 µg of intracoronary nitroglycerin injection. For IVUS, a commercially available IVUS system (iLAB, Boston Scientific, Marlborough, USA) was used for IVUS-guided RA. A 40-MHz or 60-MHz, 2.6 F imaging catheter (Boston Scientific) was located at the distal to the lesions, and automated pullback was performed at a speed of 0.5 mm/s. For OCT, A frequency-domain OCT system (ILUMIEN OPTIS, Abbott Vascular, USA) was used for OCT-guided RA. The OCT catheter (Dragonfly OPTIS, Abbott Vascular) was advanced across distal to the lesion over an angioplasty guidewire, and automatic pullback was performed at a rate of 20mm/s with continuous contrast injection to achieve a blood-free field of view.

IVUS images were analyzed using a validated software (INDEC Medical Systems, USA). Calcified plaque in IVUS images was defined as a bright echo with acoustic shadowing. OCT images were analyzed using an offline software (LightLab Imaging). Calcified plaque in OCT images was defined as a signal-poor region with sharply delineated borders. The angles of calcified plaque were analyzed using the center of mass of the lumen. All intravascular images analysis included lumen and stent area every 1 mm within the lesion and the stented segment. And all OCT and IVUS images were evaluated on the consensus of two experienced operators.

### Study endpoints and definitions

All the patients were followed up by clinical visit or telephone call. The primary endpoint was stent expansion, as previously described [9]. The secondary endpoints were the rate of procedural success and complications and the occurrence of major adverse cardiovascular events (MACE, a composite of cardiac death, myocardial infarction, target vessel revascularization and stent thrombosis) at 1-year. Severe calcified lesions were defined as radiopacities seen without cardiac motion before contrast injection usually affecting both sides of the arterial lumen, and moderate calcified lesions as radiopacities noted only during the cardiac cycle before contrast injection. The proximal and distal references were set at sites with the largest lumen area less than 20% stenosis and no major branches within 10 mm of the stenosis. Minimum stent area (MSA) was identified in each stent. Stent expansion was defined as the MSA divided by the average of the proximal and distal reference lumen areas. The procedural success was defined as a final residual stenosis < 30% after stents in the presence of TIMI3 flow.

### Statistical analysis

The statistical analysis was performed using SPSS, Version 22.0 (IBM, Armonk, New York). Continuous variables with normal distributions are presented as means ± standard deviations and were compared using an unpaired t-test. Continuous variables without normal distributions are expressed as median and first and third quartiles and were compared using the Mann-Whitney U test. Categorical variables were expressed as frequency (%) and compared with χ^2^ statistics or the Fisher exact test. Two-sided *p* < 0.05 was taken as statistical significance.

## Results

### Baseline clinical characteristics

Initially, 88 patients with coronary heart disease and moderate or severe calcified lesions treated with RA under OCT guidance or IVUS guidance in the study. However, 6 patients with no intravascular imaging data and 3 patients with in-stent restenosis lesions were excluded. Finally, 84 lesions in 79 patients (33 lesions in 32 patients in OCT-guided RA group and 51 lesions in 47 patients in IVUS -guided RA group) were included in the current study.

Baseline clinical characteristics are summarized in Table 1. There were no significant differences between OCT-guided-RA group and IVUS-guided RA group for baseline characteristics including age, gender, risk factors, comorbidities and past history of PCI and coronary artery bypass graft, and coronary heart disease diagnosis (*P* > 0.05, all).

**Table 1:** Baseline clinical characteristics

	OCT-guided RA (32 patients)	IVUS -guided RA (47 patients)	*P* value
Age	70.16 ± 10.09	68.66 ± 7.84	0.219
Male	20 (62.5%)	32 (68.1%)	0.607
BMI, kg/m^2^	25.87 ± 3.67	26.32 ± 7.95	0.708
Hypertension	23 (71.9%)	39 (83.0%)	0.238
Diabetes mellitus	17 (53.1%)	27 (57.4%)	0.704
Dyslipidemia	12 (37.5%)	18 (38.3%)	0.943
Smoking	15 (46.9%)	26 (55.3%)	0.461
LV ejection fraction, %	66.93 ± 6.91	63.89 ± 10.54	0.212
eGFR,ml/(min·1.73m^2^)	82.46 ± 20.26	80.29 ± 22.32	0.661
Previous MI	4 (12.5%)	9 (19.1)	0.434
Previous PCI	6 (18.8%)	17 (36.2%)	0.094
Previous CABG	3 (9.4%)	3 (6.4%)	0.952
*Clinical presentation*			0.577
SCAD	12 (37.5%)	14 (29.8%)	
ACS	20 (62.5%)	27 (70.2%)	

OCT, Optical coherence tomography; IVUS, Intravascular ultrasound; RA, rotational atherectomy; BMI, body mass index; LV, left ventricular; eGFR, estimated of glomerular filtration rate; MI, myocardial artery; PCI, percutaneous coronary intervention; CABG, coronary artery bypass grafting; SCAD, stable coronary artery disease; ACS, acute coronary syndrome

### Lesion and procedural characteristics

Baseline lesion and procedural characteristics were showed in Table 2. Lesions in left anterior descending artery were commonly target vessel in both OCT-guided-RA group and IVUS-guided RA group (69.7 and 64.7% respectively). There was no significant difference in the prevalence of severe calcification, lesion type, bifurcation, angulation, and multi-vessel coronary disease between the groups.

**Table 2:** Lesion and procedural characteristics

	OCT-guided RA (33 lesions)	IVUS -guided RA (51 lesions)	*P* value
*Target vessel*			
LM	1 (3.0%)	5 (9.8%)	0.457
LAD	23 (69.7%)	33 (64.7%)	0.636
LCX	1 (3.0%)	5 (9.8%)	0.457
RCA	8 (24.2%)	8 (15.7%)	0.329
Sever calcification	31 (93.9%)	48 (94.1%)	1.000
Lesion type B2/C	30 (90.9%)	48 (94.1%)	0.544
Angulation	8 (24.2%)	17 (33.3%)	0.373
Bifurcation	7 (21.2%)	17 (33.3%)	0.230
Multi-vessel coronary disease	24 (72.7%)	41 (80.4%)	0.412
Radial access	26 (78.8%)	32 (62.7%)	0.120
Initial burr size, mm	1.5 (1.5,1.5)	1.5 (1.25,1.5)	0.167
Maximal burr diameter, mm	1.5 (1.5,1.5)	1.5 (1.25,1.5)	0.290
More than 1 burr	3 (9.1%)	5 (9.8%)	0.913
Burr to artery ratio	0.55 ± 0.5	0.54 ± 0.6	0.518
Use of Scoring balloon after RA	11 (33.3%)	2 (3.9%)	0.001
Pre-dilation balloon size, mm	2.6 ± 0.3	2.7 ± 0.3	0.548
Max pre-dilation pressure, atm	17.6 ± 4.6	16.9 ± 4.4	0.471
Number of stents implanted	2.1 ± 0.6	2.0 ± 0.6	0.658
Mean stent diameter, mm	2.9 ± 0.4	3.0 ± 0.3	0.689
Total stent length, mm	63.8 ± 23.1	61.8 ± 20.5	0.526
Post-dilation balloon size, mm	3.2 ± 0.4	3.3 ± 0.4	0.714
Max post-dilation pressure, atm	20.7 ± 2.9	20.8 ± 2.9	0.811
Procedure time, min	100 (80,140)	95 (80,120)	0.515
Radiation exposure, mGy	1041 (845,1800)	1188 (853,2013)	0.618
Contrast volume, ml	348.8 ± 110.6	275.2 ± 76.8	0.002
Procedure cost, yuan	114,165 ± 31,975	107,380 ± 31,252	0.340

OCT, Optical coherence tomography; IVUS, Intravascular ultrasound; RA, rotational atherectomy; LM, left main; LAD, Left anterior descending artery; LCX, left circumflex artery; RCA, right coronary artery

Radial access was used frequently in both OCT-guided RA group and IVUS-guided RA group (78.8 and 64.7% respectively). The two groups were not significantly different with regard to the initial and maximal burr size, the burr-to-artery ratio, pre-balloon and post-balloon dilatation. The scoring balloon was more frequently performed after RA and before stenting in the OCT-guided RA group (33.3% vs. 3.9%, *P* = 0.001).

No significant difference was detected for number of stents implanted, stent diameters and length, the procedure time, radiation exposure and procedure cost in both groups. The contrast volume was significantly larger in the OCT-guided RA group [(348.8 ± 110.6) ml vs. (275.2 ± 76.8) ml, *P* = 0.002].

### Intravascular imaging findings

As shown in Table 3, the measurements of maximum calcium angle and length of calcium were not significantly different between OCT-guided-RA group and IVUS-guided RA group. OCT-guided-RA group measurements of distal reference, mean reference lumen area and lesion lumen area before stenting were smaller than IVUS-guided RA group. However, minimum stent CSA after PCI were statistically similar between OCT-guided RA group and IVUS-guided RA group (5.6 ± 1.3 mm^2^ vs. 5.7 ± 1.2 mm^2^, *P* = 0.883). Finally, there was a larger stent expansion in the OCT-guided RA group versus the IVUS-guided RA group [(82 ± 8) % vs. (75 ± 9) %, *P* = 0.001].

**Table 3:** Intravascular imaging findings

	OCT-guided RA (33 lesions)	IVUS -guided RA (51 lesions)	*P* value
Maximum calcium angle, °	360 (300,360)	360(360,360)	0.204
Length of calcium, mm	29.9 ± 14.3	25.3 ± 10.9	0.100
*Proximal reference*			
MLD, mm	2.9 ± 0.3	3.1 ± 0.3	0.015
Max LD, mm	3.4 ± 0.3	3.6 ± 0.6	0.069
lm CSA, mm^2^	8.5 ± 1.7	9.1 ± 1.8	0.131
*Distal reference*			
MLD, mm	2.3 ± 0.3	2.6 ± 0.4	0.014
Max LD, mm	2.7 ± 0.4	2.9 ± 0.3	0.005
lm CSA, mm^2^	5.3 ± 2.0	6.0 ± 1.6	0.044
Mean reference lumen CSA, mm^2^	6.8 ± 1.5	7.6 ± 1.5	0.032
Lesion MLD, mm	1.3 ± 0.3	1.5 ± 0.2	0.001
Lesion Max LD, mm	1.8 ± 0.5	1.9 ± 0.4	0.234
Lesion lumen CSA, mm^2^	1.9 ± 1.2	2.4 ± 0.6	0.043
Minimum stent area, mm^2^	5.6 ± 1.3	5.7 ± 1.2	0.883
stent expansion, %	82 ± 8	75 ± 9	0.001

OCT, Optical coherence tomography; IVUS, Intravascular ultrasound; RA, rotational atherectomy; MLD, Minimum lumen diameter; Max LD, Maximum lumen diameter; CSA, cross-sectional area

### Complications and follow-up outcomes

All of the patients in both groups achieved procedural success immediately. There were no significantly differences in the incidence of complications between groups (Table 4). No patient in either group experienced emergency CABG and cardiac tamponade during procedural. Coronary perforation occurred in 1 patient in IVUS-guided RA group and didn’t leading to a cardiac tamponade; Coronary slow flow or no-reflow occurred in 2 patients in the OCT-guided RA group and 3 patients in the IVUS-guided RA group; Coronary dissection occurred in 1 patient and 3 patients respectively in OCT-guided RA group and IVUS-guided RA group; Longitudinal stent compression occurred in 2 patients in the OCT-guided RA group, but finally we achieved favorable results with deploying an additional stent at deformed site. And contrast induced nephropathy occurred in 1 patient in OCT-guided RA group. There were no significantly differences between groups in all of this rate (*P* > 0.05, all).

**Table 4:** Complications and 1-year outcomes

	OCT-guided RA (32 patients, 33 lesions)	IVUS -guided RA (47 patients, 51 lesions)	*P* value
Procedure success	33 (100%)	51 (100%)	1.000
Complications	5 (15.2%)	7 (13.7%)	0.856
Perforation	0	1 (2.0%)	0.607
Slow flow/no reflow	2 (6.1%)	3 (5.9%)	0.973
Dissection	1 (3.0%)	3 (5.9%)	0.940
Emergency CABG	0	0	–
Longitudinal stent compression	2 (6.1%)	0	0.151
Cardiac tamponade	0	0	–
Contrast induced nephropathy	1 (3.0%)	0	0.393
MACE	1 (3.1%)	3 (6.4%)	0.517
Cardiovascular death	0	2 (4.3%)	0.351
Myocardial infarction	1 (3.1%)	0	0.405
TVR	0	1 (2.1%)	0.595
Stent thrombosis	0	1 (2.1%)	0.595

OCT, Optical coherence tomography; IVUS, Intravascular ultrasound; RA, rotational atherectomy; CABG, coronary artery bypass grafting; MACE, major adverse cardiac event; TVR, Target vessel revascularization

All the patients were followed up for 18 months (8-36months) by clinical visit or phone call. No cardiovascular death, TVR and stent thrombosis occurred in OCT-guided RA group at 1-year outcomes, but 1 patient occurred non-target vessel associated myocardial infarction. 1 patient experienced sudden death 5 days after PCI which was suspected to be stent thrombosis, 1 patient died of heart failure 1 month after discharge, and 1 patient received revascularization for restenosis of the target vessel in the IVUS-guided RA group. The frequencies of MACE, cardiovascular death, TVR and stent thrombosis at 1-year were lower in the OCT-guided RA group, but there were no significantly differences between groups (Table 4).

## Discussion

Coronary heavily calcified lesions can be a great challenge for PCI because of the strong resistance to inadequate balloon dilatation or stent deployment [10, 11]. And suboptimal stent implantation may result in in-stent restenosis and stent thrombosis [12, 13]. Thus, sufficient preparation of severely calcified lesions is particularly crucial for adequate stent expansion. RA can well modify calcified plaques and facilitate optimal stent deployment [14]. Previous study showed RA with smaller burr size (burr to artery ratio < 0.7) compared with a more aggressive strategy (burr to artery ratio > 0.7) can reduce procedural complications with similar procedural success rates [15, 16]. However, It was reported that despite the initial use of small burr,step up burr sizes was an independent indicator of MACE [17]. In contrast, additional scoring balloon use after RA was associated with lower occurrence of MACE in patients undergoing RA with a small-sized burr [18]. These studies demonstrated that application of scoring balloon to modify the calcified lesion after initial RA might be beneficial in patients with severely calcified lesions. Intravascular imaging can accurately assess calcification and outcome lesion modification, which provides important information to help operator selecting burr size, determining the endpoint of RA and guiding subsequent balloon dilatation to obtain favorable immediate effect and long-term outcomes.

Previous study had shown that minimum calcification thickness and cracks formation were associated with good stent expansion after RA [19]. And pre-dilatation with a scoring balloon resulted in better stent expansion than with conventional balloon [20]. Additionally, the best cut-off value for predicting calcification disruption was 565 μm after scoring balloon dilatation in patients with calcified coronary lesions [21]. In our study the scoring balloon was more frequently performed after RA before stent implantation in OCT-guided RA group. Since ultrasound does not penetrate calcium, calcium thickness cannot be assessed in IVUS imaging. IVUS images evaluate lesion modification after initial RA based on presence of crack and reflection or reverberation behind the calcification. But sometimes this information is not completely reliable. OCT images can assess thickness of calcification to determine the degree of lesion modification after initial RA to guide whether step up burr size or use scoring balloon dilatation or other conventional balloon for further modification. In our study OCT-guided RA obtained better stent expansion compared to IVUS-guided RA, consistent with Norihiro et al. [22] reported, but there was no frequently burr upsizing. Therefore, the present study demonstrated that under the guidance of OCT undergoing PCI combined with scoring balloon use after RA to further modify calcified lesion might be helpful to reduce burr upsizing and can also achieve larger stent expansion.

Several previous studies have shown that guidance by IVUS not only improve the immediate effect of procedural, but also improves clinical outcomes [6, 23]. And OCT-guided PCI is not inferior to IVUS-guided PCI in terms of immediate procedural result and mid-term clinical outcomes [24, 25]. In our study, both OCT-guided RA group and IVUS-guided RA group achieved procedural success immediately. There were no significantly differences in the incidence of complications. Although there was no statistical difference in the rate of MACE at 1 year between the groups, no cardiovascular death, TVR and stent thrombosis occurred in OCT-guided RA group. Hence, our study demonstrated OCT-guided RA compared to IVUS-guided RA obtained comparable immediately procedural result and may improve long term prognosis for calcified coronary lesions.

There were some limitations in our study. First, it was a single-center, retrospective study with a bit of small sample size. Second, the usage of intravascular imaging by means of IVUS and OCT were at operator’s discretion. Operators may have preferred IVUS used in patients with chronic kidney disease and left main lesion, which may have selection bias. Therefore, a larger prospective multicenter study needs to be conducted to prove the clinical impact of intravascular imaging-guided RA.

## Conclusions

Overall, our study showed that OCT-guided RA compared to IVUS-guided RA in the treatment of calcified coronary lesions achieved better stent expansion and may have improved mid-term prognosis.

## Data Availability

The datasets used and analyzed during the current study are available from the corresponding author on reasonable request.

## Abbreviations

OCT: Optical coherence tomography
IVUS: Intravascular ultrasound
RA: Rotational atherectomy
PCI: Percutaneous coronary interventions
ACT: Activated clotting time
CAG: Coronary angiography
TIMI: Thrombolysis in myocardial infarction
MACE: Major adverse cardiovascular events
CSA: Cross-sectional area
TVR: Target vessel revascularization
